# Extended-spectrum β-lactamase-producing Enterobacterales in diverse foodstuffs: a prospective, longitudinal study in the city of Basel, Switzerland

**DOI:** 10.3389/fmicb.2023.1295037

**Published:** 2023-11-22

**Authors:** Elena Gómez-Sanz, Claudia Bagutti, Ana B. García-Martín, Jan A. Roth, Monica Alt Hug, Laura Maurer Pekerman, Ruth Schindler, Reto Furger, Lucas Eichenberger, Ingrid Steffen, Philipp Hübner, Tanja Stadler, Lisandra Aguilar-Bultet, Sarah Tschudin-Sutter

**Affiliations:** ^1^Division of Infectious Diseases and Hospital Epidemiology, University Hospital Basel, University of Basel, Basel, Switzerland; ^2^Department of Clinical Research, University Hospital Basel, Basel, Switzerland; ^3^State Laboratory Basel-City, Basel, Switzerland; ^4^Rothen Laboratory, Basel, Switzerland; ^5^Department of Biosystems Science and Engineering, ETH Zurich, Zürich, Switzerland

**Keywords:** ESBL-producing Enterobacterales, foodstuff, chicken, greens, *Escherichia coli*, *Klebsiella pneumoniae*, spatiotemporal distribution

## Abstract

**Background:**

The involvement of non-human-to-human transmission of extended-spectrum β-lactamase-producing Enterobacterales (ESBL-PE) remains elusive. Foodstuffs may serve as reservoirs for ESBL-PE and contribute to their spread.

**Aim:**

We aimed to systematically investigate the presence and spatiotemporal distribution of ESBL-PE in diverse unprocessed foodstuffs of different origin purchased in a central European city.

**Methods:**

Chicken and green (herbs, salad, sprouts, vegetables) samples were collected monthly for two consecutive years, from June 2017 to June 2019, from large supermarket chains and small local food retailers, representing all ten postcode areas of the City of Basel (Switzerland), and the kitchen of the University Hospital Basel (Basel, Switzerland). After enrichment, presumptive ESBL-PE were isolated by selective culture methods and identified by Matrix-assisted laser desorption/ionization time-of-flight mass spectrometry. ESBL production was confirmed by phenotypic testing.

**Results:**

Among 947 food samples, 14.8% were positive for ESBL-PE isolate/s belonging to eight different ESBL-producing bacterial species. *Escherichia coli* and *Serratia fonticola* were predominant across samples (9 and 2%, respectively). Higher ESBL-PE prevalence was observed in chicken (25.9%) than in green (3.8%) samples (*p* < 0.001). Among greens, ESBL-PE were most frequently isolated from sprouts (15.2%). High ESBL-PE species diversity was observed among chicken samples, with *E. coli* as predominant (17.6%). ESBL-producing *Enterobacter cloacae* was detected among different greens. Yet, ESBL-producing *Klebsiella pneumoniae* was predominant in sprouts (12.1%). In total, 20.5% of samples from organic farming and 14.2% of samples from conventionally raised animals harbored an ESBL-producing isolate. Detection of ESBL-PE across samples differed between organic and non-organic when stratified by food source (*p* < 0.001), particularly among greens (12.5% organic, 2.4% conventional). High proportion of organic chicken samples was positive for ESBL-*E. coli* (33.3%), while the detection of several species characterized the conventional chicken samples. No significant differences in ESBL-PE frequences were detected between national (13.4%) and international samples (8.0%) (*p* = 0.122). Instead, differences were observed between regions of food production and countries (*p* < 0.001). No significant differences were found when comparing the proportion of ESBL-PE positive samples across districts, shop sizes and the hospital kitchen. The percentage of ESBL-PE positive samples did not differ monthly across the two-year sampling period (*p* = 0.107).

**Conclusion:**

Our findings indicate moderate dissemination of ESBL-PE in foodstuffs, especially between chicken products and sprouts. Chicken meat represents a source for several ESBL-producing Enterobacterales, especially *E. coli*, while greens are more prone to carry ESBL-*K. pneumoniae* and *E. cloacae*. We disclose the importance of food type, food production system and production origin when assessing the risk of contamination with different ESBL-PE species.

## Introduction

Antimicrobial resistance (AMR) represents a significant global health concern, demanding comprehensive understanding of its underlying sources and transmission pathways. Among the AMR contributors, extended-spectrum β-lactamase-producing Enterobacterales (ESBL-PE), particularly *Escherichia coli* and the *Klebsiella, Enterobacter, Serratia and Citrobacter* (KESC) group, have emerged as a formidable adversary, presenting challenges to infection control and therapeutic interventions (Tacconelli et al., 2018; Cassini et al., 2019; Antimicrobial Resistance Collaborators, 2022). While human-to-human transmission of ESBL-PE has been extensively studied, the extent and dynamics of non-human-to-human spread remain elusive (Ewers et al., 2012; Navon-Venezia et al., 2017; Dorado-García et al., 2018), impeding the design of effective containment strategies.

Over the last decade, ESBL-PE, mainly *E. coli*, have been increasingly identified in livestock, in the food chain, and in companion animals (Salgado-Caxito et al., 2021; Silva et al., 2023). However, the importance of these reservoirs in entertaining ongoing transmission in humans remains controversial. Increasing evidence suggests that foodstuffs might serve as potential reservoirs for ESBL-PE, acting as conduits for their dissemination throughout the food supply chain and beyond (Overdevest et al., 2014; Tschudin-Sutter et al., 2014). Among foodstuffs, poultry and chicken meat (Overdevest et al., 2011; Olsen et al., 2014; Ye et al., 2018; Economou et al., 2023), as well as vegetables, sprouts and herbs (Zurfluh et al., 2015; Ye et al., 2018; Moon et al., 2022) have been repeatedly identified as being contaminated with ESBL-PE. The high prevalence of ESBL-PE in the broiler production chain has been reported as of major concern (Projahn et al., 2018). Among meat production, poultry farming represents one of the most demanding type of animal production, rendering poultry highly susceptible to immune system weaknesses and illnesses. Furthermore, the high population densities create ideal conditions for the rapid transmission of bacteria among the flocks. While indication of clonal spread of ESBL-PE from foodstuffs to humans remains limited, ESBL-producing *E. coli* with identical genes and plasmids were detected in farm animals, foods, and humans, suggesting transmission of mobile genetic elements (Zurfluh et al., 2014, 2015; Borjesson et al., 2016; Dorado-García et al., 2018). Such an exchange of mobile genetic elements may occur in the gastrointestinal tract. Vegetables, sprouts and herbs may present particularly important reservoirs as they may be consumed raw, thus pass high concentrations of resistance genes into the human intestine (Holzel et al., 2018).

These foodborne ESBL-PE bacteria may not only compromise the safety of the food supply chain but also exacerbate the spread of ESBL-PE into human populations (Arvand et al., 2015; Aguilar-Bultet et al., 2021).

To address this pressing knowledge gap, we performed a systematic prospective investigation with the aim to discern the presence and spatiotemporal distribution of ESBL-PE in different unprocessed foodstuffs, focusing on chicken, herbs, sprouts, salads and vegetables originating from several sources in a central European city.

## Materials and methods

### Study design, setting and data collection

#### Study design and setting

This prospective and cross-sectional longitudinal study (Stadler et al., 2018) (pre-registered on ClinicalTrials.gov; identifier NCT03465683) was performed over a 24-month period in Basel, Switzerland, a central European medium size city with a population of around 180′000 inhabitants (June 2022). Fresh food products distributed across the city retailers/markets were targeted.

Food samples were collected monthly for two consecutive years (from June 2017 to June 2019). Ten postal code areas distributed across the city were targeted every month. For this, two retailers per postal code area (except for 4059 with one) were planned to be sampled at each sampling round. Shops were chosen to achieve a representative distribution of large retailers, butchers and small local shops (including market stalls). Some shops were alternatively sampled each month (depending on even and odd numbered month) to achieve best possible coverage of different shop types across the city. One chicken and one piece of greens were collected each round. For small shops/market stalls, in most cases, two stores were needed to purchase both types of food (Supplementary Table S1). In total, 31 different retailers were sampled along the study period. During each sampling round, a single sample of food per food source (chicken and greens) was also collected from the kitchen of the University Hospital Basel (Basel, Switzerland). As a result, a total of 40 samples were collected per sampling round.

#### Definitions and data collection

All food samples were categorized as chicken or greens (main category). The term “greens” includes all fresh unwashed herbs, sprouts, salads and vegetable samples. “Chicken” refers to different chicken parts, ranging from chicken breast, minced to stewing hen. Food types were further classified as chicken, herbs, sprouts, salads, and vegetables. The term “large retailer” refers to major supermarket chains with multiple branches or large department stores that featured food departments. The term “small retailer” was assigned to local specialized stores, local market stalls or shops with a couple of local branches, mostly offering one food type. Information about the food production system (conventional or organic farming) was recorded. The regional origin of each food sample was registered and geographic regions were classified as follows: Central Europe (Austria, Germany, Hungary, Slovenia, Switzerland, Switzerland/Morocco), Western Europe (France, The Netherlands), Southern Europe (Italy, Italy/France, Italy/Hungary, Spain), Eastern Europe (Bulgaria, Ukraine), North Africa (Morocco), Eastern Africa (Ethiopia, Kenia), Southern Africa (South Africa), Oriental Asia (China), South Asia (India), Southeast Asia (Thailand, Vietnam), Middle East (Israel), South America (Brazil). Presumptive ESBL-PE refer to Enterobacterales species recovered from selective plating with 3^rd^ geneneration cephalosporin (see below) prior to resistance confirmation testing. ESBL-PE refer to phenotypically confirmed ESBL-producing Enterobacterales. Calendar months span from January (1) to December (12).

### Food sample collection, processing and isolation of presumptive ESBL-PE

Samples were aseptically collected and kept at 4°C till processed (maximum 3 days after sampling). For this, 10 g of the chicken and green samples were weighed out and placed in a sterile Stomacher bag. An enrichment step was performed for both sample sources to increase sensitivity, hence, to recover as many and phenotypically diverse ESBL-PE as possible. For the chicken samples, 90 mL of Enterobacteriaceae Enrichment (EE) Broth (Oxoid, Thermo Fisher Diagnostics, Reinach, Switzerland) was added. For greens, 90 mL of Buffered Peptone Water (BPW) was added. All samples were then individually homogenised in a Stover for 1 min before being incubated overnight at 37°C. The enrichments of the chicken samples were streaked on chromogenic Brilliance™ ESBL plates (Oxoid) the following day using an inoculation loop to obtain single colonies. The plates were then incubated overnight at 37°C and photographed the following day. Three millilitres of the greens cultures were taken and added to 30 mL of EE broth, enhanced with 1 mg/L cefotaxime (a 3^rd^ generation cephalosporin), and incubated overnight at 37° C. On the following day, the greens enrichments were streaked on chromogenic Brilliance™ ESBL plates (Oxoid) plates in the same way as the chicken enrichments and incubated overnight at 37°C. The presumptive identification of ESBL-PE was based on the colour and morphology of colonies (ESBL-producing *E. coli* (blue/pink) and the KESC group (green)) grown on the chromogenic Brilliance™ ESBL plates. One to three colonies per colour and/or morphology were chosen and further isolated on new Brilliance™ ESBL plates.

### Species identification, ESBL confirmation and selection criteria

Species identification of all isolates was performed in duplicate on fresh cultures by Matrix-Assisted Laser Desorption/Ionization Time-Of-Flight mass spectrometry (MALDI-TOF MS). The colonies were fixed on the MALDI-TOF MS plate using a CHAC matrix (alpha-cyano-4-hydroxycinnamic acid) (Ziegler et al., 2015) and processed using the MALDI-TOF MS AximaTM Confidence (Shimadzu-Biotech, Reinach, Switzerland) and the SARAMIS™ Database (Spectral Archive And Microbial Identification System, AnagnosTec, Potsdam-Golm, Germany) (Welker and Moore, 2011). Both readouts needed to be consistent for bacterial-species taxonomy assignment. Therefore, no clear identification applied when either both measurements were ambiguous (i.e., below 75% identification) or one of the two duplicates gave no result. If no clear identification was achieved, the spectra were matched against the ribosomal marker-based database PAPMID™ (Mabritec AG, Riehen, Switzerland) (Kassim et al., 2017) applying the same performance criteria. Isolates identified as Enterobacterales and forming colonies of different colour and/or morphology were stored in glycerol/Triptic Soy Broth (TSB) (1:1) at −80°C for downstream analyses. Isolates not identified as Enterobacterales, those with unclear or inconsistent identification, and duplicates with the same identification as other selected isolates per sample were discarded and considered as not fulfilling the study inclusion criteria. It should be noted that our MALDI-TOF database at the time of identification could not discriminate some species of the *K. pneumoniae* species complex (including *K. quasipneumoniae*, *K.* var*iicola* and *K. quasivariicola*). Likewise, *E. cloacae* isolates may include other species of the complex, such as *E. ludwigii* and *E. kobei*, as they were not included in the database (Gómez-Sanz et al., 2023).

ESBL confirmatory testing was performed and evaluated on all stored isolates according to the Clinical & Laboratory Standards Institute (CLSI) guidelines (CLSI, 2013) and using the Total ESBL Confirm Kit (Rosco Diagnostica, Axon Lab, Baden, Switzerland). The type of cephalosporin tested (cefotaxime and ceftazidime, cefepime) depended on the species and was applied according to the manufacturer’s instructions. For *Raoultella* spp., ascribed to the genus *Klebsiella* until 2001 (Appel et al., 2021), cefotaxime and ceftazidime were used. The plates were incubated at 35°C for 18 ± 2 h. ESBL production was confirmed when the zone of inhibition of the disk with cephalosporine plus clavulanate was ≥5 mm larger than the one around the cephalosporine alone. All isolates fulfilling these criteria were designated as ESBL-PE.

### Statistical analyses

Microbiological and food/retail characteristics were assessed both on an isolate-and collapsed sample-level. Sample-level analyses were conducted taking into account the total number of samples collected (n = 947). Isolate-level analyses were performed considering only the number of individual bacterial isolates collected (n = 1,321). Respective differences in distribution were analysed with Chi-squared/Fisher’s exact tests or Wilcoxon rank sum/Kruskal-Wallis tests – as appropriate and computationally feasible given the number of strata. Missing data is indicated throughout. We plotted matrix information with heatmaps (Stata packages “heatplot” (Jann, 2019), “palettes” and “colrspace”). Intracluster correlation was marginal overall and was therefore not considered for the explorative hypothesis tests. All analyses and visualizations were performed on a multicore system with Stata/MP, version 16 (Stata Corp., College Station, Texas, United States). All reported *p*-values are two-sided and were considered significant at value of *p* <0.05.

## Results

### ESBL-PE positive sample confirmation and ESBL-PE species identification

Out of 960 scheduled food samples, 947 (471 chicken and 476 greens) were collected across the two-year sampling period (13 samples could not be collected due to shop closures).

In total, 1,321 presumptive ESBL-PE isolates were recovered (Supplementary Table S2). Of these, 314 met the study inclusion criteria. ESBL production was phenotypically confirmed in 52.2% of the analysed isolates (164/314). ESBL-PE isolates were recovered from 14.8% (140/947) of all food samples (Tables 1, 2). The majority of samples (85.7%, 120/140) yielded one ESBL-PE isolate. Only 20 samples yielded more than one ESBL-PE isolate. Specifically, two ESBL-PE isolates were found in 17 samples (12.1%), three in two samples (1.4%) and four in one sample (0.8%) (Supplementary Table S2).

**Table 1:** Abundance of extended-spectrum β-lactamase-producing Enterobacterales (ESBL-PE) species across samples tested (*n* = 946) and isolates recovered (*n* = 1,321).

ESBL-producing species	Samples		Isolates	
	No. ESBL positive	ESBL positive (%)	No. ESBL positive	Relative abundance (%)
*Escherichia coli*	87	9.2	100	61.0
*Serratia fonticola*	20	2.1	22	13.4
*Klebsiella pneumoniae*	14	1.5	15	9.1
*Serratia liquefaciens*	13	1.4	13	7.9
*Enterobacter cloacae*	5	0.5	5	3.0
*Serratia* sp.	4	0.4	4	2.4
*Fam. Enterobacteriaceae*	2	0.2	2	1.2
*Enterobacter xiangfangensis*	1	0.1	1	0.6
*Moellerella wisconsensis*	1	0.1	1	0.6
*Proteus mirabilis*	1	0.1	1	0.6
Total	148^a^	15.6	164	100.0

a In total, 140 food samples were positive for ESBL. Of these, eight samples enclose more than one species. Four samples enclosed ESBL-producing *E. coli* and *K. pneumoniae*; one sample ESBL-producing *E. coli* and a *Serratia* sp. isolate; one ESBL-producing *E. coli* and a Fam. Enterobacteriaceae isolate; one ESBL-producing *E. cloacae* and *E. xiangfangensis*; and one ESBL-producing *P. mirabilis* and *S. fonticola*.

**Table 2:** Abundance of food products carrying extended-spectrum β-lactamase-producing Enterobacterales (ESBL-PE) and ESBL-producing isolates recovered.

Food characteristics			Samples			Isolates		
Source	Type	Product^a^	No. recovered	No. ESBL-positive	%	No. recovered	No. ESBL-positive	%
Chicken	Chicken	Chicken (breast)	293	80	27.3	397	96	24.2
		Chicken (leg)	105	23	21.9	137	23	16.8
		Chicken (wings)	38	11	28.9	56	11	19.6
		Chicken (mince)	20	6	30.0	26	8	30.8
		Stewing hen	12	1	8.3	16	1	6.3
		Corn fowl	1	1	100.0	1	1	100.0
		*Others*	2	0	0.0	2	0	0.0
Greens	Herbs	Coriander	41	4	9.8	67	9	13.4
		Basil	36	1	2.8	51	1	2.0
		Rocket	20	1	5.0	34	1	2.9
		Water spinach	3	1	33.3	8	1	12.5
		Sage	8	1	12.5	11	1	9.1
		Pakwan	1	1	100.0	2	1	50.0
		*Others*	235	0	0.0	325	0	0.0
	Salad	Lettuce (lambs)	23	2	8.7	37	2	5.4
		*Others*	50	0	0.0	49	0	0.0
	Sprouts	Sprouts (alfalfa)	7	2	28.6	9	2	22.2
		Sprouts (mung bean)	5	2	40.0	10	3	30.0
		Sprouts (radish)	5	1	20.0	8	1	12.5
		*Others*	16	0	0.0	25	0	0.0
	Vegetables	Spring onion	12	1	8.3	18	1	5.6
		Mung bean	2	1	50.0	4	1	25.0
		*Others*	12	0	0.0	28	0	0.0
		Total	947	140		1,321	164	

a “*Others*” refer to food products that tested ESBL negative (Chicken = 1; Herbs = 29; Salad = 10; Sprouts = 4; Vegetables = 4).

Eight ESBL-PE species belonging to the Enterobacterales order were identified (Table 1). However, six isolates could not be identified to the species level. Of these, four ESBL-PE isolates were classified as *Serratia* spp., and two isolates were assigned to the Enterobacteriaceae family. *Escherichia coli* was predominant across samples (9.2%), accounting for 61.0% of all ESBL-PE isolates (100/164), followed by *Serratia fonticola* (2.1%), *Klebsiella pneumoniae* (1.5%) and *Serratia liquefaciens* (1.4%) (Table 1). The proportion of positive phenotypic ESBL confirmation tests differed significantly across species (*p* < 0.001; Supplementary Figure S1), ranging from >99% for *E. coli* (100/101), *K. pneumoniae* (15/15), *Proteus mirabilis* and *Moellerella wisconsensis* (1 each); 76.5% for *Serratia* spp. isolates (39/51) (*S. fonticola*, *S. liquefaciens, Serratia* sp.), to 25% for *Enterobacter xiangfangensis* (1/4) and 7.1% for *Enterobacter cloacae* (5/70).

### ESBL-PE distribution across food types and products

Sixty-five different food products were tested at different frequencies (Supplementary Table S3). Of these, 18 products were sporadically or reiteratively positive for ESBL-PE. Table 2 displays the abundance of food products positive for ESBL-PE across all samples and isolates recovered in this study. Minced chicken, mung sprouts and mung beans were the food products with the highest ESBL-PE prevalence across chicken and greens (30.0, 40.0, 50.0%, respectively).

**Figure 1:**
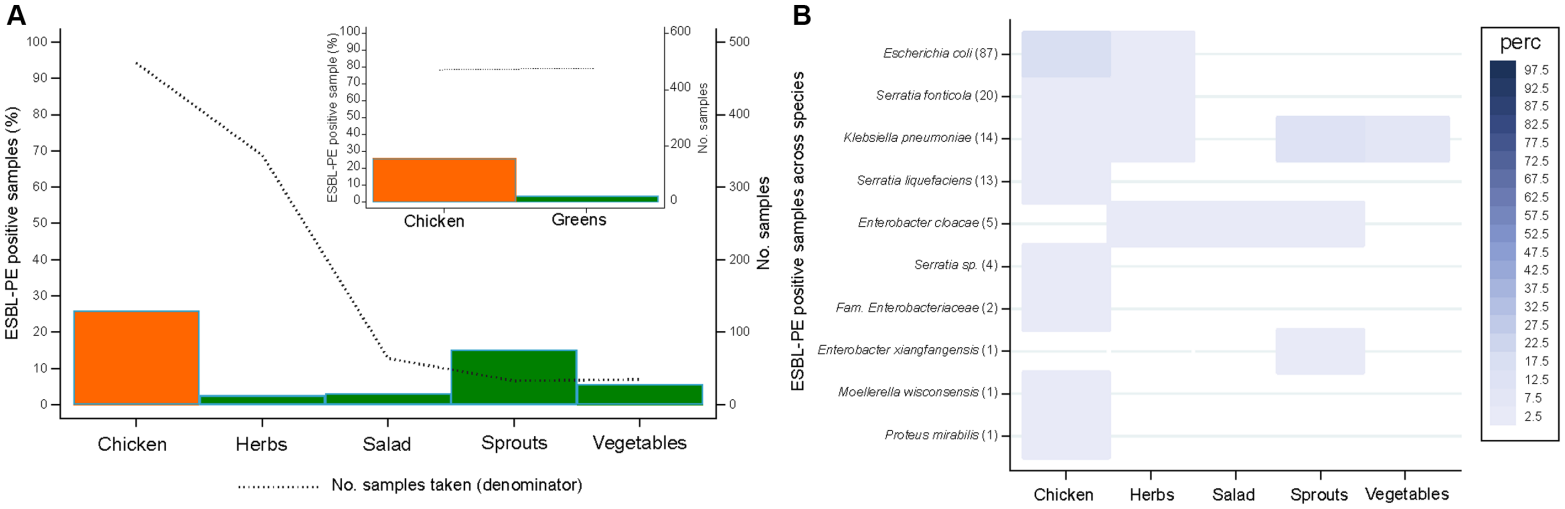
Recovery of extended-spectrum β-lactamase-producing Enterobacterales (ESBL-PE) **(A)** and distribution of ESBL-producing species **(B)** across samples for different food types. Species are ordered according to the number of ESBL samples per species (in brackets). Only species with ≥1 ESBL-PE isolate are represented. Number of samples tested per food type: 471 (chicken), 344 (herbs), 64 (salad), 33 (sprouts), 35 (vegetables).

The percentage of food samples yielding ESBL-PE differed between chicken (25.9%, 122/471) and greens (3.8%, 18/476) (*p* < 0.001). Among greens, ESBL-PE were most frequently isolated from sprouts (15.2%, 5/33) (Figure 1A).

Chicken samples enclosed six of the eight ESBL-PE species identified in this study, the species most commonly identified being *E. coli* (17.6%, 83/471). Lower species diversity was detected among greens (Figure 1B). Of these, herbs and sprouts enclosed ESBL-producing *E. coli* and/or several KESC group species at low rates (≤ 3% of samples, per species) except for *K. pneumoniae* (12.1%, 4/33). Salad and vegetables exclusively harbored ESBL-producing *E. cloacae* (3.1%, 2/64) or *K. pneumoniae* (5.7%, 2/35), respectively. *K. pneumoniae* was the most dispersed ESBL species, as it was detected in chicken and all greens but salad, followed by *E. cloacae*, present in all greens except for vegetables.

### ESBL-PE distribution across food production systems

**Figure 2:**
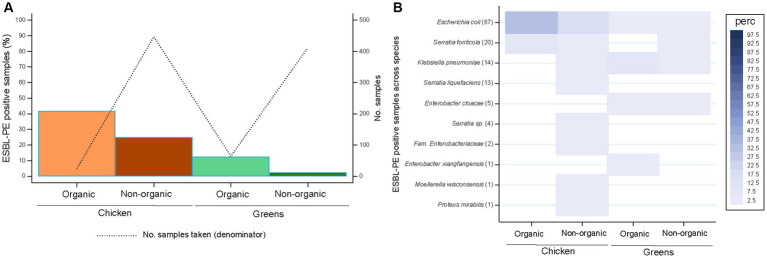
Recovery of extended-spectrum β-lactamase-producing Enterobacterales (ESBL-PE) **(A)** and distribution of ESBL-producing species **(B)** across samples per food production system stratified by food source. Species are ordered according to the number of ESBL-PE samples per species (in brackets). Only species with ≥1 ESBL-PE isolate are represented. Number of samples tested per production system stratified by food source: 24 (organic chicken), 447 (non-organic chicken), 64 (organic green), 412 (non-organic green).

Most samples originated from conventional farming (*n* = 859), while 88 samples derived from organic farming (Figure 2A). All organic products came either from Switzerland (21 chicken, 53 greens) or EU countries (3 chicken samples [France], 11 from greens [3 Germany, 4 Italy, 1 Spain, 3 multiple origins]). In total, 122 (14.2%) samples from conventional farming and 18 (20.5%) from organic production were positive for ESBL-PE (*p* = 0.116). In contrast, the percentage of chicken and greens samples positive for ESBL-PE differed significantly between organic and non-organic farming (*p* < 0.001). Specifically, significant differences were noticed for greens from organic production in comparison with conventional farming (12.5% versus 2.4%, respectively, *p* = 0.001; Figure 2A).

The ESBL-PE sample distribution per country or region of production stratified by food source and food production system is shown in Supplementary Tables S4, S5, respectively. Organic samples containing ESBL-PE accounted for 38.1% (8/13) and 15.1% (8/45) of Swiss organic chicken and greens, respectively. These originated from seven of 15 Swiss retailers from which organic samples were collected. In addition, ESBL-PE were found in 66.7% (2/3) of French organic chicken samples. Both ESBL-PE samples from French organic chicken were obtained from one large retailer but collected in two different years (August 2017 and October 2018) (Supplementary Table S2). All but one retailer with ESBL-PE positive organic samples were classified as large.

ESBL-producing *E. coli* was the single species present across all four food groups (i.e., both food categories and food production systems; Figure 2B). Organic chicken samples yielded only ESBL-producing *E. coli* and *S. fonticola*, but at approximately double the rate as in the non-organic counterparts (33.3% versus 16.8%; 8.3% versus 3.8%, respectively). Instead, non-organic chicken was characterised by broad ESBL-producing species diversity at low rates (< 4%). ESBL-producing *E. coli*, *K. pneumoniae* and *E. cloacae* were present in both organic and non-organic greens, but at remarkable higher rates across organic greens (1.6% versus 0.7%; 7.8% versus 1.2%; 3.1 versus 0.7%, respectively).

### ESBL-PE distribution across geographic regions of food production

**Figure 3:**
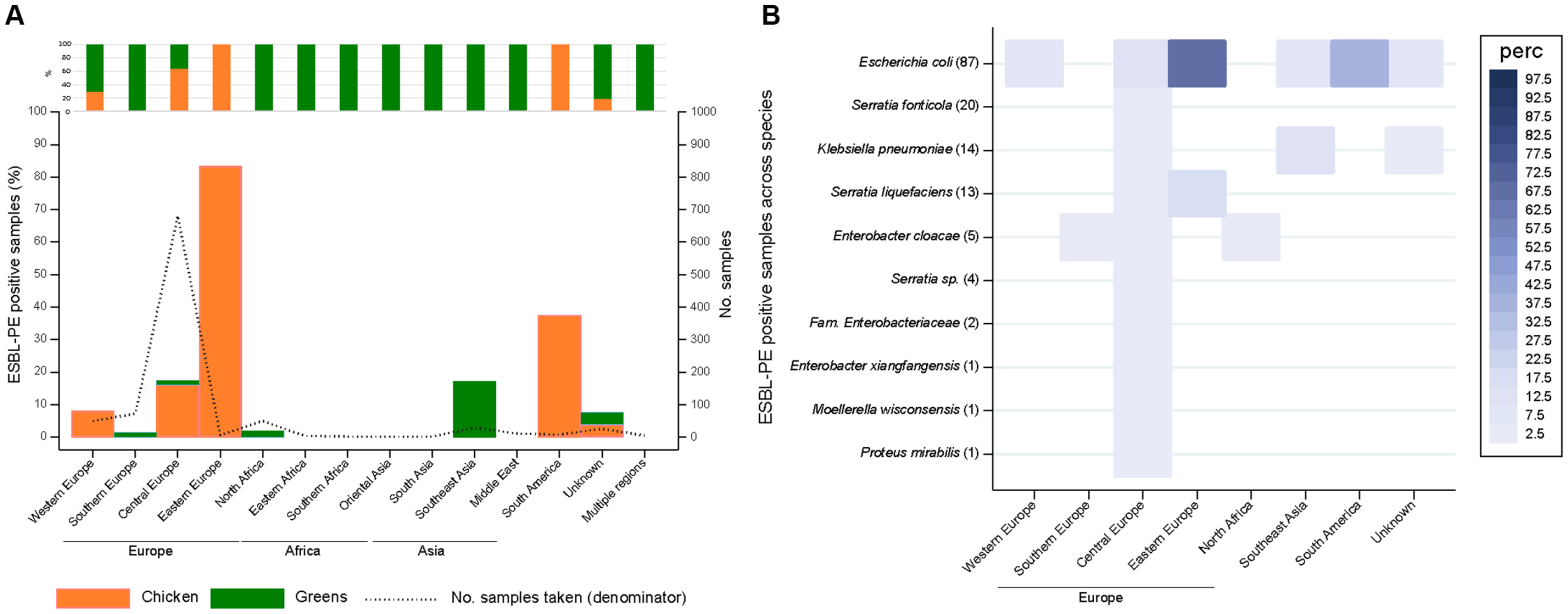
Recovery of extended-spectrum β-lactamase-producing Enterobacterales (ESBL-PE) **(A)** and distribution of ESBL-producing species **(B)** across samples per geographic region stratified by food source (for Figure 3A). **(A)** The upper stacked bar chart denotes the distribution of samples taken per food source across geographic regions. **(B)** Species are ordered according to the number of ESBL-PE samples per species (in brackets). Only species with ≥1 ESBL-PE isolate are represented. Number of analysed samples per geographic region: 50 (Western Europe), 72 (Southern Europe), 681 (Central Europe), 6 (Eastern Europe), 50 (North Africa), 4 (Eastern Africa), 2 (Southern Africa), 1 (Oriental Asia), 2 (South Asia), 29 (Southeast Asia), 11 (Middle East), 8 (South America), 26 (unknown), 5 (multiple origins).

A total of 610 samples originated from Switzerland and 311 were imported from different countries. Eastern European and South American countries were exclusively supplying chicken. Instead, all African and Asian samples consisted of greens. The only countries contributing both chicken and greens samples were from Western and Central Europe (in addition to 21 green and 5 chicken products of unknown origin; Figure 3A upper pannel).

The overall ESBL-PE frequency irrespective of the food category across national versus foreign products showed no significant differences (13.4% versus 18.0%, *p* = 0.122) (Supplementary Table S6). In contrast, the percentage of samples positive for ESBL-PE differed significantly across countries (Supplementary Figure S2) as well as across region of food production (Figure 3A; *p* < 0.001).

The ESBL-PE sample distribution per country or region of production across food source is shown in Supplementary Tables S7, S8, respectively. When stratified by Swiss and foreign meat, 19.5% of the Swiss samples were positive versus 50.5% among foreign countries. However, this trend was not observed follow when focusing on ESBL-producing *E. coli* (4.5% versus 4.6%). No significant differences were noted in the ESBL-PE occurrence of national versus imported fresh greens (4.2% versus 3.3%, respectively).

The highest percentage of ESBL-PE was detected for samples originating from Eastern Europe (83.3%, 5/6 chicken), followed by South America (37.5%, 3/8 chicken) (Figure 3A). ESBL-PE rates across greens were considerably higher (17.2%; 5/29) among samples from Southeast Asia compared to other greens suppliers.

Overall, the distribution of ESBL-producing species differed across the geographic regions (*p* = 0.025; Figure 3B). Highest possible ESBL-producing species diversity was observed across samples from Central Europe, although at low percentages. This is in line with the fact that 72% (681/947) of samples originated from this geographic region. The rate of ESBL-producing *E. coli* from Eastern European and South American samples was particularly high (66.7%, 4/6 and 37.5%, 3/8, respectively). ESBL-producing *K. pneumoniae* was more prevalent among greens from Southeast Asia (13.8%, 4/29) than from the other positive regions.

### Spatiotemporal distribution of ESBL-PE across samples from small/large retailers and the kitchen of the University Hospital Basel

**Figure 4:**
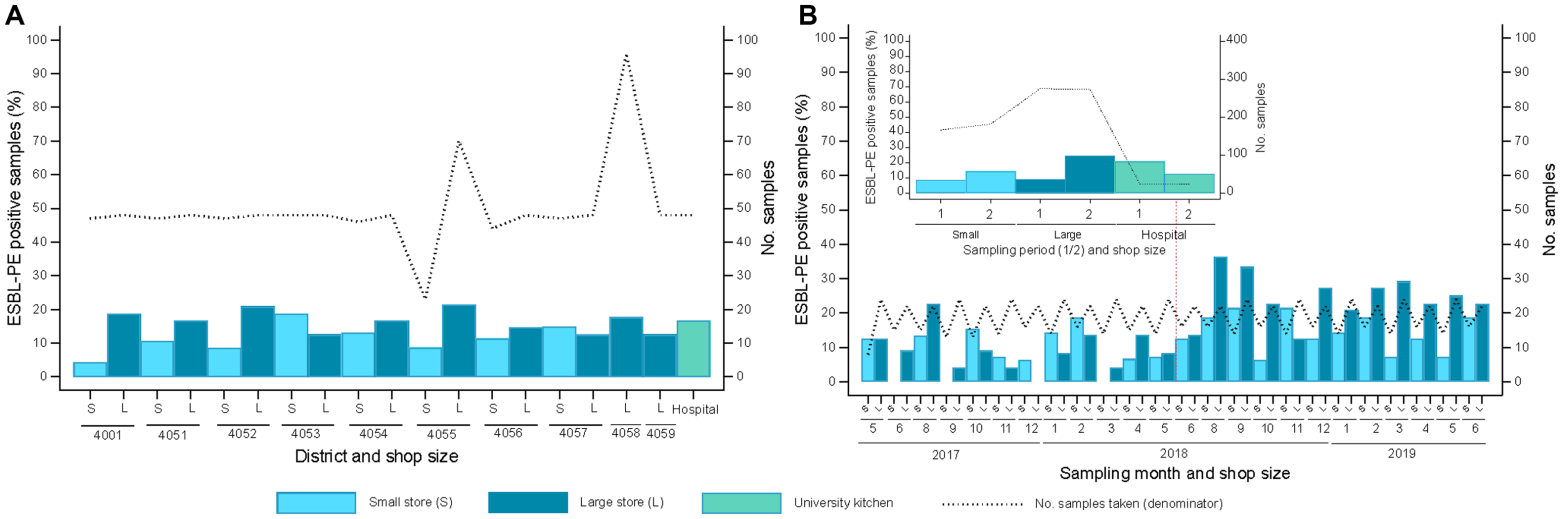
Spatiotemporal distribution of extended-spectrum β-lactamase-producing Enterobacterales (ESBL-PE) samples stratified per shop size (S, small; L, large) and the hospital kitchen. **(A)** Spatial distribution of samples with ESBL isolates/s categorized by large and small shop per district (20 districts, 31 shops plus USB kitchen). District distribution is as follows (number of shops tested per shop size – small/large): 4001 (2/3), 4051 (0/3, plus hospital kitchen), 4052 (2/1), 4053 (0/2), 4054 (2/1), 4055 (2/2), 4056 (2/1), 4057 (1/2), 4058 (0/4) 4059 (0/1). The upper embedded graph displays the compiled ESBL spatial distribution per shop size and hospital kitchen samples. **(B)** Distribution of ESBL-positive samples across sampling months (natural calendar month) clustered by shop size (16 small, 15 large). The upper embedded graph displays the compiled ESBL temporal distribution stratified by shop size and sampling period (1, samplings 1–12, 2, samplings 13–24).

The 31 different shops sampled along the study period were categorized into small (n = 16) and large retailers (*n* = 15) (Supplementary Table S1). At least one ESBL-PE-positive sample was collected from 26 of the 31 retailers (including all 15 large retailers and 11 of 16 small retailers) plus the hospital kitchen. The proportion of samples positive for ESBL-PE showed no significant differences across districts and shop sizes (*p* = 0.720). Yet, large shops tended to exhibit a higher percentage of ESBL-PE isolates (small: 11.5%; large: 16.7%; *p* = 0.084). ESBL-PE were detected in 16.7% of samples from the hospital kitchen (Figure 4A).

Overall, the rate of ESBL-PE positive samples did not differ monthly across the two-year sampling period (*p* = 0.107). Yet, ESBL-PE positive samples increased in the second sampling year, for both small (14.3% versus 8.4%) and large retailers (24.5% versus 9.1%) (*p* < 0.001) (Figure 4B).

**Figure 5:**
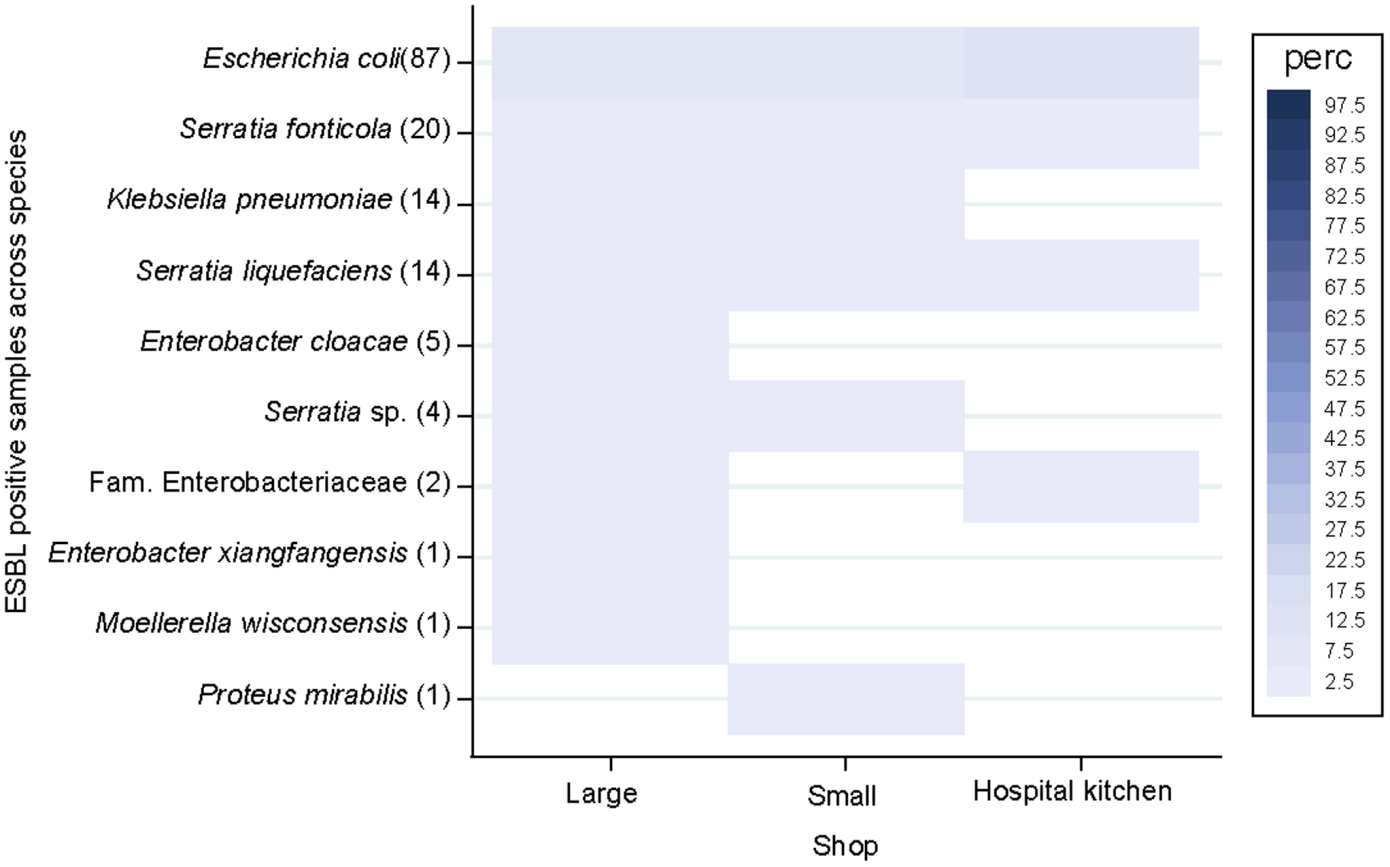
Extended-spectrum β-lactamase-producing Enterobacterales (ESBL-PE) species distribution of samples across shop size categories and the hospital kitchen. Species are ordered according to the number of ESBL-PE samples per species (number of ESBL samples within brackets). Only species with ≥1 ESBL-PE isolate are represented. Number of analysed samples per stratum: 550 (large), 349 (small), 48 (hospital kitchen).

ESBL-producing species distribution did not differ across the clustered shop sizes and the hospital kitchen (*p* = 0.055; Figure 5). ESBL-PE rates were < 3% across all species but *E. coli* (8.3–12.5%). ESBL-producing *E. coli*, *S. fonticola* and *S. liquefaciens* were detected in all three supplier groups. ESBL-producing *E. cloacae*, *E. xiangfangensis* and *M. wisconsensis* were only sporadically detected in samples from large market chains, while ESBL-producing *P. mirabilis* was only detected in one sample from a small local retailer.

## Discussion

More than every tenth samples were found ESBL-PE positive among diverse foodstuffs in the city of Basel over a two-year study period. Highest levels were detected in chicken (25.9%) and sprouts (15.2%). Our data support the importance of food type, food production system and production origin when assessing the risk of contamination with different ESBL-PE species and highlights that consumers may be exposed through the food chain to ESBL-PE, posing a hazard to food safety and public health.

The overall ESBL-PE prevalence observed among chicken meat samples from all origins (25.9%) is in line with rates of ESBL/AmpC-producing *E. coli* reported in the ANRESIS (Swiss Centre for Antibiotic Resistance) surveillance report in Switzerland in 2020 (29.4%) (Federal Office of Public Health and Federal Food Safety and Veterinary Office, 2022).^1^ When stratified by Swiss and foreign meat, 10.2% of the Swiss samples were positive versus 61.8% among foreign countries (EU member states). While the overall ESBL-PE proportions we observed among national versus imported retail chicken meat follow a similar tendency (19.5% versus 50.5%, respectively), we did not observe this trend when focusing on ESBL-producing *E. coli* (4.5% versus 4.6%; overall 9.2%). Notably, the ANRESIS report observes a steady decrease in ESBL/AmpC-producing *E. coli* since 2014 in both domestically produced chicken meat and meat from abroad (42% among Swiss chicken meat in 2016, 21% in 2018; 10% in 2020 as compared to >60% in 2014, versus 65% in chicken meat from abroad in 2016, 63% in 2018, 62% in 2020 as compared to >80% in 2014) (Federal Office of Public Health and Federal Food Safety and Veterinary Office, 2022). Former European studies report higher ESBL-PE values (75–94%) (Leverstein-van Hall et al., 2011; Kaesbohrer et al., 2019; Randall et al., 2021).

In contrast, we did not observe differences in the ESBL-PE occurrence of national versus imported fresh greens (4.2% versus 3.3%, respectively), and the overall prevalence was low (3.8%). These values are notably lower than those reported by Zurfluh and colleagues (Zurfluh et al., 2015) for vegetables imported into Switzerland (25.4%) or those reported from the Netherlands for sprouts collected between 2013 and 2016 (19%) (Huizinga et al., 2018). They detected ESBL-producing *E. coli* as predominant species while *K. pneumoniae* was dominant among our samples, followed by *E. cloacae* (ESBL-producing *K. pneumoniae* 10/947; ESBL-producing *E. cloacae* 5/947; ESBL-producing *E. coli* 4/947).

All ESBL-PE recovered in our study belonged to either the Enterobacteriaceae (*E. coli*, *K. pneumoniae*, *Serratia* spp., *Enterobacter* spp.) or Morganellaceae (*M. wisconsensis* and *P. mirabilis*) families. ESBL-producing *E. coli* was the most common species, mirroring the epidemiology of ESBL-PE in clinical (Gasser et al., 2019; Antimicrobial Resistance Collaborators, 2022) and wastewater samples (Zaatout et al., 2021; Gómez-Sanz et al., 2023). Notably, we observed mainly ESBL-producing *E. coli* among organic chicken (33.3%) versus high species diversity among samples from conventionally bred chicken. This could be due to a more clonal spread among organic chicken isolates.

ESBL-producing *S. fonticola* was the second most common species detected (2% of all samples). It was recovered from chicken (both organic and conventional) and non-organic greens. *S. fonticola* was recently reported as the most common ESBL-producing species recovered from fresh vegetables collected in Spain (Pintor-Cora et al., 2021) and fresh spinach from South Africa (Richter et al., 2021) supporting that vegetables can serve as important reservoirs for antibiotic resistance genes.

For some ESBL-producing species, such as *E. coli*, *K. pneumoniae* or *Serratia* spp., the selective media Brilliance™ ESBL agar, a validated commercial selective agar used for the identification of presumptive ESBL-producing *E. coli* and KESC group isolates (Blane et al., 2016; Lee et al., 2021), turned out to be an excellent or good predictor for ESBL-producing isolates (> 99, 77%, respectively). However, for other species, particularly for *Enterobacter* spp., only 8% of them were phenotypically confirmed as ESBL positive. Using the same media, Blane and colleagues (Blane et al., 2016) observed that pre-enrichment with cefpodoxime significantly increased sensitivity (from 59% to up to 98%) but reduced selectivity (from 87% to up to 61%), due to increased growth of non-ESBL-PE isolates. These results support that our pre-enrichment-based isolation method was appropriate in prioritizing sensitivity and species diversity, while increasing the range of false positive isolates, which were efficiently discriminated using the Rosco ESBL confirmation test.

We observed higher ESBL-PE percentages in samples from organic versus conventionally raised products, both in chicken meat and greens samples. A recent study from Spain supports organic fresh fruits and vegetables as important reservoirs for β-lactam-and even carbapenem-resistant bacteria (Jimenez-Belenguer et al., 2023). Comparable differences in detection rates of ESBL-PE between organic and non-organic grown vegetables have also been previously reported from the Netherlands (van Hoek et al., 2015), and between organic and non-organic chicken meat samples from Germany (Kola et al., 2012) and the Netherlands (Cohen Stuart et al., 2012). However, not all studies show this trend. Our results are in contrast to previous studies reporting higher proportions of ESBL-PE in chicken from conventional farming as compared to chicken from organic farming (Uyanik et al., 2021).

Another study comparing AMR among *E. coli* in the fecal microbiota of young calves raised in organic and in conventional dairy farms in Switzerland, found AMR to be highly prevalent irrespective of the farm management system, with proportions of certain resistance phenotypes higher among organic calves (Nuesch-Inderbinen et al., 2022). In contrast, the occurrence of ESBL producers among young dairy calves was possibly linked to factors associated with conventional farming (Nuesch-Inderbinen et al., 2022). These differences favoring ESBL-PE recovery from conventional farming as compared to organic farming have been explained by conventional farming practices being related to the use of antimicrobials and close contact among animals and a potentially contaminated environment for most of the production cycle (Collignon et al., 2013; Musa et al., 2020).

The high contamination rate of organic meat could at first sight be surprising because of the limited use of antibiotics in organic farm during the rearing process, as antibiotics may be considered an important reason for the occurrence of ESBL-producing strains in chickens. A possible explanation could be the introduction of ESBL-PE-colonized one-day-old broilers into the organic farms. Roger Stephan and coworkers provided evidence for a vertical transmission of *bla*_CTX-M-1_-carrying resistant plasmids within the broiler production pyramid without antimicrobial selection pressure (Zurfluh et al., 2014). Alternatively, cross contamination may occur between conventional and organic flocks in the slaughter plants, as has been reported for *Salmonella* (Rasschaert et al., 2007), or at the retail level. In addition, soil and surface water contaminated by ESBL-PE (Blaak et al., 2015) or carrying environmental ESBL-PE might serve as a source of contamination of organically grown chickens due to their higher exposure to the environment. Remarkably, the use of therapeutic antimicrobials may be higher in organic poultry related to the renunciation of in-feed coccidiostats, as previously suggested by a Norwegian report (Norwegian Scientific Committee for Food and Environment (VKM), 2020). Coccidiostats are used to prevent Coccidiosis but may also prevent some bacterial infections, such as necrotic colitis, caused by *Clostridium perfringens*.

We acknowledge that conclusions based on food products should be interpreted with caution. Cross contaminations during processing and handling (Cohen Stuart et al., 2012; Mollenkopf et al., 2018) as well as the exposure of humans may influence the ESBL-PE prevalence. Overall, the stated potential contamination sources for organic products, while possible, require further investigation.

No significant differences in ESBL-PE abundance were detected between national (13.4%) and foreign samples (8.0%). Instead, variances were observed between regions of food production and countries, potentially reflecting differences in production and antibiotic use across countries. We acknowledge, however, that our sample size for individual countries is too small to draw valid conclusions regarding geographic differences.

This study has some eminent limitations. First, the number of samples collected per category (such as food type, country of origin and production type) was limited and unbalanced, thus impeding generalizability of our results. Second, we do not have additional information on the food managing practises apart from the “Bio” labels of the organic products. This limits the interpretation of the influence of the farming system on our results as we miss important information that may help explain the differences observed between organic and conventional samples for both chicken and greens. Likewise, the historical and rearing practises of chicken (specially along the food pyramid) and agricultural crops may differ considerably. Third, exposure of both animals (especially organic) and the fresh produce to environmental sources of antimicrobial and/or antimicrobial resistance determinants (such as wildlife, irrigation water, organic fertilizers, etc) is not known. Fourth, antibiotic use along the production system could not be testified. Fifth, our exploratory comparisons and associated hypothesis tests should not be considered as confirmatory. Overall, an even sample distribution in terms of food product, origin and source, recording external environmental factors, and a comprehensive exploration of food management and rearing practices reveals crucial to confirm the differential patterns observed here. On top, processing and distribution steps are additional contamination sources that may be relevant, since contamination close to consumption may inherently be more likely to result in human exposure. Yet, our work covers one of the largest study periods and applies one of the more consistent sampling efforts as compared to former studies on ESBL-PE detection in foodstuffs. Additionally, we reveal potential critical factors to consider when designing future studies on ESBL-PE sources from foodstuffs.

This study forms part of a bigger effort to study the prevalence of ESBL-PE in the city of Basel, measured by detection in wastewater (Gómez-Sanz et al., 2023), and to study their possible transmission between food and humans, for which clinical samples were recovered in the city in the same period (Stadler et al., 2018). Thanks to this strenuous sampling effort and subsequent comprehensive analyses, we could identify a plausible food-to-human transmission event, which involved one of the chicken breast meat isolates recovered here (conventional farming) and two clinical isolates from patients admitted to the University Hospital Basel (Aguilar-Bultet et al., 2021).

In conclusion, our findings indicate moderate dissemination of ESBL-PE in foodstuffs, especially raw chicken products and sprouts. Fresh chicken represents a source for different ESBL-producing species, especially *E. coli*, while greens are more prone to carry ESBL *K. pneumoniae* (specially sprouts) and *E. cloacae*. Our results disclose the importance of food type, food production system (organic, conventional) and production origin when assessing the risk of contamination with different ESBL-PE species. Consistent and rigorous methodologies are essential to yield findings that can shape public health policies effectively. The metadata recorded in this study suggests critical categories to consider for future study designs addressing the attribution of different sources of ESBL-PE from foodstuffs to the general population. We highlight that consumers are exposed to ESBL-PE species through the food chain, posing a hazard to food safety and public health. Systematic surveillance of ESBL-PE in foodstuffs is crucial for early detection of community-based sources of ESBL-PE.

## Data availability statement

The original contributions presented in the study are included in the article/Supplementary material, further inquiries can be directed to the corresponding author.

## Author contributions

EG-S: Formal analysis, Methodology, Investigation, Writing – original draft. CB: Formal analysis, Investigation, Conceptualization, Supervision, Writing – review & editing. AG-M: Writing – review & editing. JR: Writing – review & editing, Formal analysis. MA: Writing – review & editing, Investigation. LP: Investigation, Writing – review & editing. RS: Investigation, Writing – review & editing, Project administration. RF: Investigation, Writing – review & editing. LE: Investigation, Writing – review & editing. IS: Investigation, Writing – review & editing. PH: Investigation, Resources, Writing – review & editing. TS: Methodology, Writing – review & editing. LA-B: Data curation, Investigation, Writing – review & editing. ST-S: Conceptualization, Formal analysis, Funding acquisition, Methodology, Project administration, Resources, Supervision, Writing – review & editing.

## Footnotes

^1^ https://www.anresis.ch/publication-category/sarr/
