# Supplementary material for: Extended-spectrum β-lactamase-producing Enterobacterales in diverse foodstuffs: a prospective, longitudinal study in the city of Basel, Switzerland
Source: Front Microbiol. 2023 Nov 22;14:1295037. doi: 10.3389/fmicb.2023.1295037 (PMC10703160; doi:10.3389/fmicb.2023.1295037)
Supplement: Supplementary file 2 [file Data_Sheet_1.pdf]

Supplementary Figures

Extended-spectrum β-lactamase-producing Enterobacterales in diverse foodstuffs: a prospective, longitudinal study in the City of Basel, Switzerland

Elena Gómez-Sanz[1,2]†, Claudia Bagutti[3]†, Ana B. García-Martín[1,2], Jan A. Roth[1], Monica Alt[3], Laura Maurer Pekerman[1,2], Ruth Schindler[1,2], Reto Furger[3], Lucas Eichenberger[3], Ingrid Steffen[4], Philipp Hübner[3], Tanja Stadler[5], Lisandra Aguilar-Bultet[1,2], Sarah Tschudin-Sutter[1,2*]

[1] Division of Infectious Diseases and Hospital Epidemiology, University Hospital Basel, University of Basel, Basel, Switzerland.

[2] Department of Clinical Research, University Hospital Basel, Basel, Switzerland.

[3] State Laboratory Basel-City, Basel, Switzerland.

[4] Rothen Laboratory, Basel, Switzerland.

[5] Department of Biosystems Science and Engineering, ETH Zurich, Zurich, Switzerland.

†These authors contributed equally to this work

***Correspondence**: sarah.tschudin@usb.ch

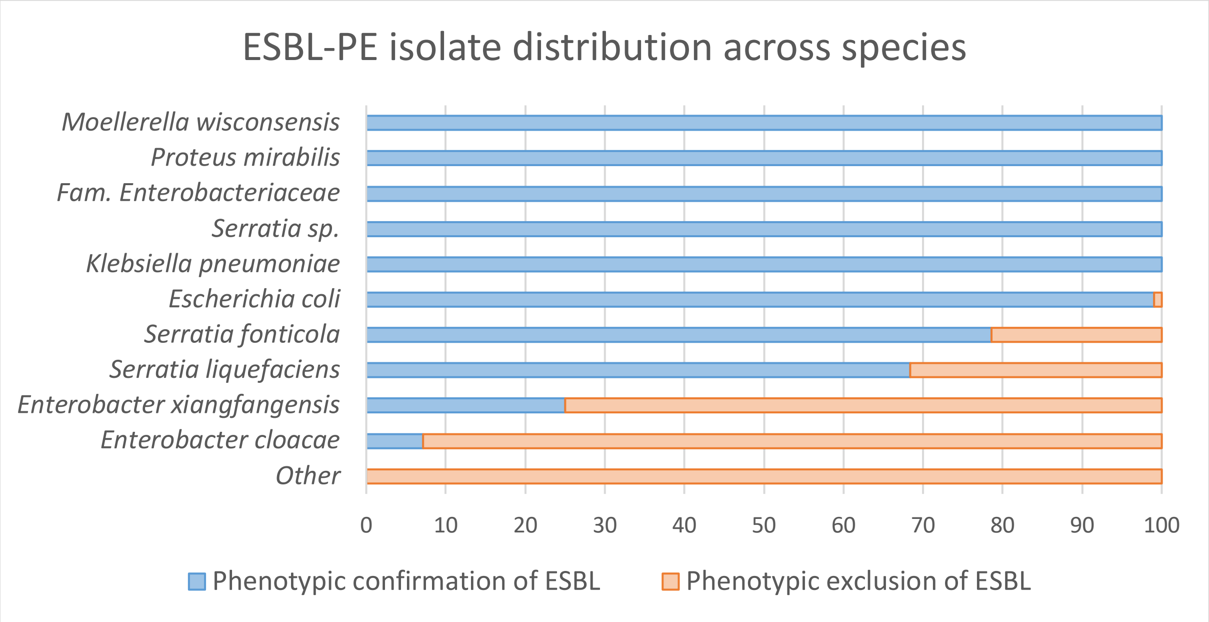

Supplementary Figure S1. Phenotypic Extended-Spectrum β-lactamase (ESBL) distribution across the tested Enterobacterales (n = 314).

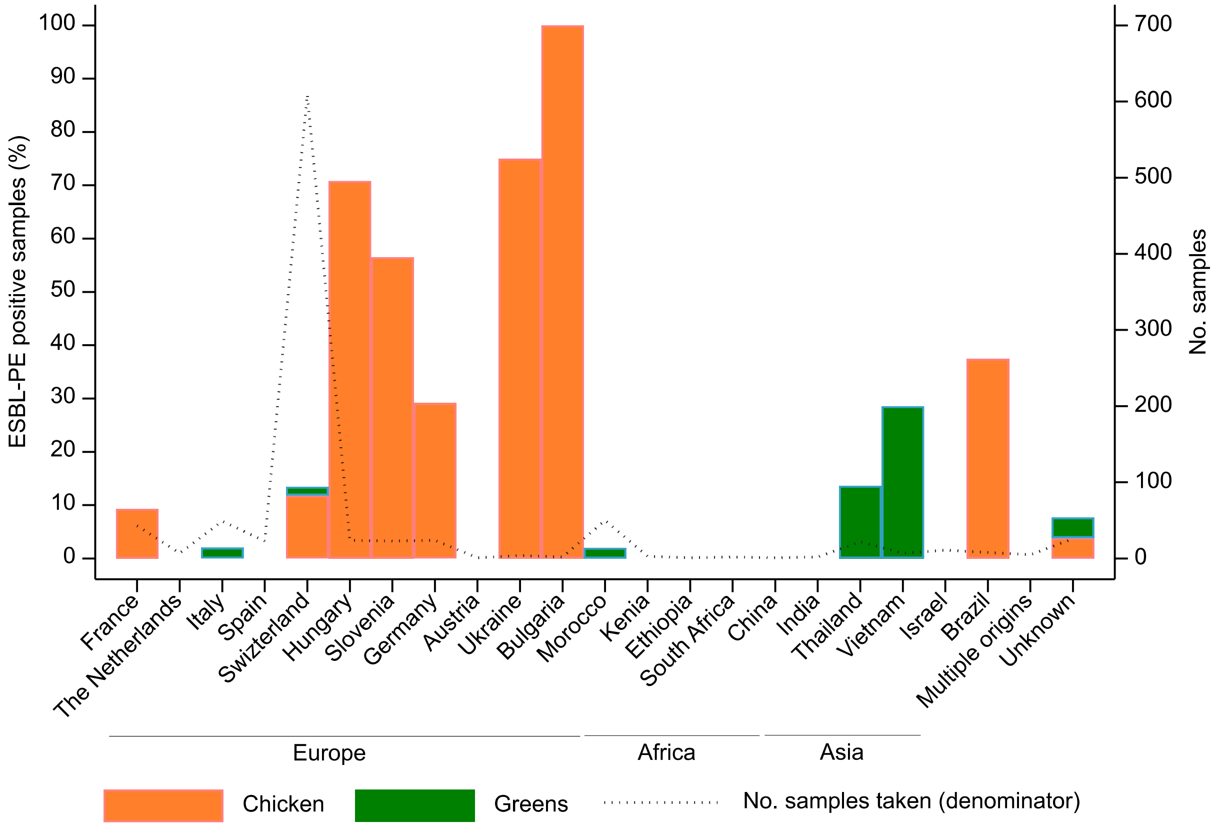

Supplementary Figure S2. Spatial distribution of Extended-Spectrum β-lactamase Producing Enterobacterales (ESBL-PE)-containing samples stratified per country of food production.
